
JOURNAL INFORMATION
==============================
NLM Title Abbreviation: Orthop Surg
Journal ID: Orthop Surg
NLM Journal ID: 101501666
Journal ID: OS

Orthopaedic Surgery

ISSN: 1757-7853
EISSN: 1757-7861
Publisher: Wiley

ARTICLE INFORMATION
==============================
PMCID: PMC11474517
DOI: 10.1111/os.13781
Article ID: OS13781
Article version: 1
Subjects: Issue Information, Issue Information

Issue Information

Electronic publication date: 2024 Jan 11
Publication date: 2024 Jan
Volume: 16
Issue: 1
First page: 1
Last page: 2
Copyright: © 2024 The Authors. Orthopaedic Surgery published by Tianjin Hospital and John Wiley & Sons Australia, Ltd.
License: This is an open access article under the terms of the http://creativecommons.org/licenses/by/4.0/ License, which permits use, distribution and reproduction in any medium, provided the original work is properly cited.
License URL: https://creativecommons.org/licenses/by/4.0/

Figure count: 0
Table count: 0
Page count: 6

source-schema-version-number: 2.0
cover-date: January 2024
details-of-publishers-convertor: Converter:WILEY_ML3GV2_TO_JATSPMC version:6.4.9 mode:remove_FC converted:15.10.2024

==============================

Volume 16 • Issue 1 • January 2024 • ISSN 1757-7861

### Orthopaedic Surgery

Open Access

REVIEW ARTICLE

3
KINESITHERAPY FOR KNEE OSTEOARTHRITIS PATIENTS PHYSICAL AND PSYCHOLOGICAL HEALTH BASED ON "TRADITIONAL CHINESE EXERCISE" MANAGEMENT MODALITIES: A SYSTEMATIC REVIEW AND META-ANALYSIS OF RANDOMIZED CONTROLLED TRIALS

CLINICAL ARTICLES

17
COMPARISON OF LONG-TERM FOLLOW-UP OF N-HA PA66 CAGE AND PEEK CAGE OF LUMBAR INTERBODY FUSION IN MULTI-LEVEL DEGENERATIVE LUMBAR DISEASES: A STEPWISE PROPENSITY SCORE MATCHING ANALYSIS

29
UNRELIABILITY OF SERUM- OR PLASMA-BASED ASSAYS OF D-DIMER OR FIBRIN (FIBRINOGEN) DEGRADATION PRODUCT FOR DIAGNOSING PERIPROSTHETIC JOINT INFECTION: A PROSPECTIVE PARALLEL STUDY

38
ADMISSION INFLAMMATION MARKERS INFLUENCE LONG-TERM MORTALITY IN ELDERLY PATIENTS UNDERGOING HIP FRACTURE SURGERY: A RETROSPECTIVE COHORT STUDY

47
A NOVEL MULTIPLE SCREW DISTRACTION REDUCER SYSTEM IN THE TREATMENT OF SCOLIOSIS WITH A SEVERE RIB HUMP

57
THE QUALITY OF LIFE OF THE PATIENTS WITH THORACIC OSSIFICATION OF THE LIGAMENTUM FLAVUM AFTER SURGERY USING EQ-5D-5L

72
POSTOPERATIVE PREVALENCE AND RISK FACTORS FOR SERUM HYPOKALEMIA IN PATIENTS WITH PRIMARY TOTAL JOINT ARTHROPLASTY

78
SURGICAL STRATEGY FOR RECURRENT GIANT CELL TUMOR IN THE THORACOLUMBAR SPINE

86
BONE HEALING AND CLINICAL OUTCOME FOLLOWING MEDIAL OPENING-WEDGE HIGH TIBIAL OSTEOTOMY USING WEDGE-SHAPED CANCELLOUS ALLOGRAFT

94
PREDICTING UNION, OSTEOMYELITIS, AND AMPUTATION OUTCOMES OF GUSTILO IIIC OPEN TIBIAL FRACTURES: A RETROSPECTIVE STUDY

104
POSSIBLE MECHANISM AND TREATMENT RESULTS OF COMBINED PEDIATRIC FRACTURES OF THE HUMERAL LATERAL CONDYLE AND IPSILATERAL ULNAR OLECRANON

111
DEVELOPMENT AND VALIDATION OF A RISK-PREDICTION NOMOGRAM FOR PREOPERATIVE BLOOD TYPE AND ANTIBODY TESTING IN SPINAL FUSION SURGERY

123
DO PATIENTS WITH BORDERLINE ANEMIA NEED TREATMENT BEFORE TOTAL HIP ARTHROPLASTY? A PROPENSITY SCORE-MATCHED COHORT STUDY

132
INTRODUCTION AND AN ANALYSIS OF INTER- AND INTRA-OBSERVER VALIDITY TO THE CLASSIFICATION OF HOFFA-LIKE TIBIAL PLATEAU FRACTURES

140
KIRSCHNER WIRE INTERNAL FIXATION OF THE MEDIAL TIBIOTALAR JOINT FOR INDIRECT REPAIR OF DELTOID LIGAMENT INJURY: A RETROSPECTIVE COMPARATIVE STUDY

RESEARCH ARTICLES

149
PREDICTORS ASSOCIATED WITH FORGOTTEN KNEE IN PATIENTS WITH TOTAL KNEE ARTHROPLASTY BASED ON MULTIVARIABLE LINEAR REGRESSION

157
A CADAVERIC STUDY OF THE OPTIMAL ISOMETRIC REGION ON THE ANTEROLATERAL SURFACE OF THE KNEE IN ANTEROLATERAL LIGAMENT RECONSTRUCTION

167
TGF-β1 INHIBITS OSTEOCLAST DIFFERENTIATION AND ABNORMAL ANGIOGENESIS IN INTERVERTEBRAL DISC DEGENERATION: EVIDENCE FROM RNA SEQUENCING AND ANIMAL STUDIES

183
DISCOVERY OF TAUROCHOLIC ACID SODIUM HYDRATE AS A NOVEL REPURPOSING DRUG FOR INTERVERTEBRAL DISC DEGENERATION BY TARGETING MAPK3

196
AUTOMATIC DETECTION AND CLASSIFICATION OF MODIC CHANGES IN MRI IMAGES USING DEEP LEARNING: INTELLIGENT ASSISTED DIAGNOSIS SYSTEM

207
EXCESS MORTALITY FOR FEMORAL INTERTROCHANTERIC FRACTURE PATIENTS AGED 50 YEARS AND OLDER TREATED SURGICALLY AND CONSERVATIVELY IN TIANJIN, CHINA: A COHORT STUDY

216
RACE AND GENDER DIFFERENCES IN ANTERIOR CRUCIATE LIGAMENT FEMORAL FOOTPRINT LOCATION AND ORIENTATION: A 3D-MRI STUDY

227
INCREASED EXPRESSION OF INFLAMMATORY CYTOKINES AND DISCOGENIC NECK PAIN

TRADITIONAL CHINESE MEDICINE IN ORTHOPAEDICS

234
FACTORS ASSOCIATED WITH RE-DISPLACEMENT AFTER NONSURGICAL TREATMENT OF DISTAL RADIUS FRACTURES IN ADULTS: A RETROSPECTIVE STUDY

OPERATIVE TECHNIQUES

245
A FEMORAL NECK OSTEOTOMY FOR THE PATIENTS WITH ANKYLOSING SPONDYLITIS AND THORACOLUMBAR KYPHOSIS COMBINED WITH HIP FLEXION CONTRACTURE

254
ARTHROSCOPIC BONE GRAFTING AND ROBOT-ASSISTED FIXATION FOR SCAPHOID NONUNION

263
MINIMALLY INVASIVE TREATMENT OF INVERSION SHORTENING CALCANEAL FRACTURES IN THE "OUT-IN" POSITION

269
MINIMALLY INVASIVE PERCUTANEOUS LEVERAGE REDUCTION IN THE TREATMENT OF PEDIATRIC SALTER–HARRIS IV AND V DISTAL FEMORAL PHYSEAL FRACTURES

BRIEF REPORT

276
LIAW'S ELLIPSE ANTEVERSION METHOD FOR DISTINGUISHING ACETABULAR COMPONENT RETROVERSION FROM ANTEVERSION ON PLAIN RADIOGRAPHS

CASE REPORT

282
LOCALLY INVASIVE PIGMENTED VILLONODULAR SYNOVITIS OF THE WRIST TREATED BY ARTHROSCOPIC RESECTION AND BONE GRAFTING

WILEY

View this journal online at wileyonlinelibrary.com/journal/os

### Orthopaedic Surgery

**Editor-in-Chief**
Gui-xing Qiu, *Beijing, China*
Francis John Hornicek, *Boston, USA*

**Associate Editor**
Yong-cheng Hu, *Tianjin, China*
Thomas Blattert, *Leipzig, Germany*
Douglas Letson, *Tampa, USA*
Xin-long Ma, *Tianjin, China*
Ling Qin, *Hong Kong, China*

**Editorial Board**
Giovanni Alessi, *Gent, Belgium*
Haluk Berk, *Izmir, Turkey*
Thomas Blattert, *Leipzig, Germany*
Mathis Bostrom, *New York, USA*
Kao-wha Chang, *Taiwan, China*
Edmund YS Chao, *Baltimore, USA*
Liao-bin Chen, *Wuhan, China*
Yan-xi Chen, *Shanghai, China*
Kenneth MC Cheung, *Hong Kong, China*
Thomas L. Clemens, *Birmingham, USA*
Benny Thellefsen Dahl, *Copenhagen, Denmark*
Lian-fu Deng, *Shanghai, China*
Jean Destandau, *Bordeaux, France*
Jian Dong, *Shanghai, China*
Shun-wu Fan, *Hangzhou, China*
David Fang, *Hong Kong, China*
Shi-jun Gao, *Shijiazhuang, China*
Wei Guo, *Beijing, China*
Yong Hai, *Beijing, China*
Alan R. Hargens, *San Diego, USA*
Francis John Hornicek, *Boston, USA*
William C. Horton, *Atlanta, USA*
John Hsiang, *Seattle, USA*
Yong-cheng Hu, *Tianjin, China*
Qing Jiang, *Nanjing, China*
Paul Jutte, *Groningen, Netherlands*
Jean Pierre Kalala, *Gent, Belgium*
Choon-Ki Lee, *Seoul, Korea*
Jean Jacque Lehuec, *Bordeaux, France*
Lawrence G. Lenke, *Seattle, USA*
Douglas Letson, *Tampa, USA*
Kwok Sui Leung, *Hong Kong, China*
Jian-hao Lin, *Beijing, China*
Bao-ge Liu, *Beijing, China*
Wei-hee Lo, *Taiwan, China*
William Weijia Lu, *Hong Kong, China*
Keith DK Luk, *Hong Kong, China*
Zong-ping Luo, *Suzhou, China*
Cyril Mauffrey, *Denver, USA*
Wei-hu Ma, *Ningbo, China*
Xin-long Ma, *Tianjin, China*
Ling Qin, *Hong Kong, China*
Gui-xing Qiu, *Beijing, China*
Axel Reinhardt, *Potsdam, Germany*
Pu-yi Sheng, *Guangzhou, China*
Harry L Shufflebarger, *Miami, USA*
David Stulberg, *Chicago, USA*
Paul Sung, *San Diego, USA*
Paul Unwin, *Leicester, UK*
Luc F. De Waele, *Gent, Belgium*
Fei Wang, *Shijiazhuang, China*
Wei-jun Wang, *Nanjing, China*
James N. Weinstein, *Chicago, USA*
Hee-Kit Wong, *Singapore*
Su-jia Wu, *Nanjing, China*
Jian-ru Xiao, *Shanghai, China*
Hui-lin Yang, *Suzhou, China*
Tzai-chiu Yu, *Taiwan, China*
Xi-jie Yu, *Chengdu, China*
Hansen A. Yuan, *New York, USA*
Hong-wen Zhu, *New York, USA*

**Address for Editorial Correspondence:** Editorial Board of *Orthopaedic Surgery*, Tianjin Hospital, 122 Munan Road, Tianjin 300050, China (email: orthopaedicsurgery@126.com; telephone: +86 22 2833 4734; fax: +86 22 2824 1184).

**Disclaimer:** The Publisher, Tianjin Hospital and Editors cannot be held responsible for errors or any consequences arising from the use of information contained in this journal; the views and opinions expressed do not necessarily reflect those of the Publisher, Tianjin Hospital and Editors, neither does the publication of advertisements constitute any endorsement by the Publisher, Tianjin Hospital and Editors of the products advertised.

**Copyright © 2024 The Authors. Orthopaedic Surgery published by Tianjin Hospital and John Wiley & Sons Australia, Ltd**

**Copyright and Copying (in any format)**
Copyright © 2024 The Authors. Orthopaedic Surgery published by Tianjin Hospital and John Wiley & Sons Australia, Ltd. All rights reserved. No part of this publication may be reproduced, stored or transmitted in any form or by any means without the prior permission in writing from the copyright holder. Authorization to copy items for internal and personal use is granted by the copyright holder for libraries and other users registered with their local Reproduction Rights Organisation (RRO), e.g. Copyright Clearance Center (CCC), 222 Rosewood Drive, Danvers, MA 01923, USA (www.copyright.com), provided the appropriate fee is paid directly to the RRO. This consent does not extend to other kinds of copying such as copying for general distribution, for advertising or promotional purposes, for republication, for creating new collective works or for resale. Permissions for such reuse can be obtained using the RightsLink "Request Permissions" link on Wiley Online Library. Special requests should be addressed to: permissions@wiley.com

For submission instructions, subscription and all other information visit: http://wileyonlinelibrary.com/journal/os

ISSN 1757-7861 (Online)

OS.JEB.January24
